# The Role of the Right Hemisphere in Processing Phonetic Variability Between Talkers

**DOI:** 10.1162/nol_a_00028

**Published:** 2021-02-01

**Authors:** Sahil Luthra

**Affiliations:** Psychological Sciences, University of Connecticut, Storrs, CT, USA

**Keywords:** speech perception, talker identity, vocal identity, phonetic variability, right hemisphere, functional neuroimaging

## Abstract

Neurobiological models of speech perception posit that both left and right posterior temporal brain regions are involved in the early auditory analysis of speech sounds. However, frank deficits in speech perception are not readily observed in individuals with right hemisphere damage. Instead, damage to the right hemisphere is often associated with impairments in vocal identity processing. Herein lies an apparent paradox: The mapping between acoustics and speech sound categories can vary substantially across talkers, so why might right hemisphere damage selectively impair vocal identity processing without obvious effects on speech perception? In this review, I attempt to clarify the role of the right hemisphere in speech perception through a careful consideration of its role in processing vocal identity. I review evidence showing that right posterior superior temporal, right anterior superior temporal, and right inferior / middle frontal regions all play distinct roles in vocal identity processing. In considering the implications of these findings for neurobiological accounts of speech perception, I argue that the recruitment of right posterior superior temporal cortex during speech perception may specifically reflect the process of conditioning phonetic identity on talker information. I suggest that the relative lack of involvement of other right hemisphere regions in speech perception may be because speech perception does not necessarily place a high burden on talker processing systems, and I argue that the extant literature hints at potential subclinical impairments in the speech perception abilities of individuals with right hemisphere damage.

## INTRODUCTION

A rich neuroscientific literature has established the importance of the brain’s left hemisphere for processing language. Early patient data demonstrated that damage to left superior temporal (Wernicke, 1874) and left inferior frontal (Broca, 1861) brain regions can lead to a loss of language abilities (i.e., aphasia), and recent studies also support a critical role for left hemisphere structures in the process of speech perception specifically. In particular, a wealth of neuroimaging evidence suggests that left superior temporal regions are important for imposing category structure on acoustically similar speech sounds (Desai et al., 2008; Liebenthal et al., 2010; Luthra, Guediche, et al., 2019; Mesgarani et al., 2014; Myers, 2007; Yi et al., 2019) and that left inferior frontal regions play a key role in differentiating between similar speech sound categories (Lee et al., 2012; Myers, 2007; Myers, Blumstein, et al., 2009; Rogers & Davis, 2018; Xie & Myers, 2018).

Relatively less is known about the extent to which the right hemisphere plays a role in speech perception, which may largely be a result of the fact that damage to the right hemisphere does not typically result in an aphasia (Blumstein & Myers, 2014; Turkeltaub & Branch Coslett, 2010). Instead, research on the right hemisphere’s role in language processing has largely focused on its high-level role in processing pragmatic information (Siegal et al., 1996) such as emotional prosody (Heilman et al., 1984), metaphorical language (Schmidt et al., 2007), and other forms of nonliteral language, including humor and sarcasm (Mitchell & Crow, 2005). While prominent neurobiological models (e.g., the Dual Stream Model; Hickok & Poeppel, 2000, 2004, 2007) have proposed at least some degree of right hemisphere involvement in processing phonetic information, the precise function of the right hemisphere in speech perception is relatively underspecified, especially compared to the more detailed characterization of the left hemisphere.

Notably, however, the right hemisphere has been heavily implicated in vocal identity processing—that is, in processing perceptual information about a voice in order to identify who is talking (Maguinness et al., 2018; Perrodin et al., 2015). Neuropsychological studies have linked right hemisphere strokes to deficits in identifying people by voice (Luzzi et al., 2018; Roswandowitz et al., 2018; Van Lancker & Canter, 1982; Van Lancker & Kreiman, 1987), though strikingly, patients with right hemisphere damage do not typically show frank deficits in speech perception. It is puzzling that these patients show deficits in vocal identity processing but not in speech perception, since talker processing and phonetic processing are known to be closely tied; the mapping between acoustic information and phonetic information can vary considerably across talkers, and theoretical accounts of speech perception argue that to perceive the speech signal accurately, listeners condition phonetic identity on talker information (Johnson, 2008; Joos, 1948; Kleinschmidt, 2019; Kleinschmidt & Jaeger, 2015). Given that phonetic processing is tightly linked to talker information, I suggest that by considering the role of the right hemisphere in processing nonlinguistic information about vocal identity, we might better understand the role of the right hemisphere in speech perception.

Note that in this review, I use the term “talker processing” largely to refer to the processing of voice information in support of processing speech, consistent with the use of the term “talker” in the speech perception literature. In contrast, I use “vocal identity processing” to refer to the processing of voice information to determine who is talking. These two processes are assumed to be theoretically distinct but to rely on some shared cognitive and neural architecture (Maguinness et al., 2018).

The structure of this review is as follows. After briefly discussing the interdependence between phonetic processing and talker processing, I review the existing literature on the role of the right hemisphere in vocal identity processing, paying careful attention to the contributions of different brain regions. I then consider current perspectives on the role of the right hemisphere in speech perception before closing with the hypothesis that the right hemisphere (and the right superior posterior temporal cortex in particular) may play an important role in allowing listeners to condition phonetic identity on talker information during speech perception.

### How Is Phonetic Processing Linked to Talker Processing?

Individual talkers can differ substantially in how they produce their speech sounds, with talkers varying both In their use of rapid temporal cues such as voice-onset time (VOT; Allen et al., 2003) and in their use of spectral cues that indicate phoneme identity (Peterson & Barney, 1952). A vast literature indicates that listeners are highly sensitive to these talker-specific differences in phonetic variation and that they adjust the mapping between acoustic information and phonetic categories accordingly (e.g., Allen & Miller, 2004; Clayards et al., 2008; Kraljic & Samuel, 2005; Norris et al., 2003; Theodore & Monto, 2019). More generally, theoretical accounts of speech perception posit that listeners maintain distinct sets of beliefs about how different talkers produce their speech sounds (Kleinschmidt & Jaeger, 2015), meaning that phonetic processing is intrinsically linked to talker information.

The interdependence between phonetic processing and talker processing is further highlighted by studies showing that phonetic processing is facilitated when listeners are familiar with a particular talker (a talker familiarity effect) and by studies showing that talker processing is facilitated when listeners are familiar with the phonetic inventory of a particular language (a language familiarity effect). With regard to the former, several studies have found that talker familiarity leads to perceptual gains when processing speech in noise (Kreitewolf, Mathias, & von Kriegstein, 2017; Nygaard & Pisoni, 1998; Souza et al., 2013), and that talker familiarity makes it easier to selectively attend to one talker while ignoring another (Holmes et al., 2018; Holmes & Johnsrude, 2020; Johnsrude et al., 2013; Newman & Evers, 2007). With regard to the language familiarity effect, a number of studies have demonstrated that talker identification is facilitated when listeners hear speech in their native language (in which they are familiar with the phonetic category structure) compared to when they hear speech in a foreign language (in which they are not; Goggin et al., 1991; Perrachione & Wong, 2007). Talker familiarity effects can be understood by considering that when listeners receive practice with a particular talker, the acoustic dimensions that are relevant for processing that talker’s voice acquire distinctiveness; if the same dimensions are relevant for both talker processing and phonetic processing, then experience with a talker should incur performance benefits for phonetic processing (Nygaard & Pisoni, 1998). Similarly, language familiarity effects can be understood by recognizing that when listeners are familiar with the phonetic inventory of a particular language, the key acoustic-phonetic dimensions for that language likewise acquire distinctiveness—and if the same dimensions are relevant for talker processing, then experience with phonetic processing should yield benefits for talker processing. Taken together, such findings indicate that speech perception and talker processing are highly interrelated processes.

### How Does the Right Hemisphere Support Vocal Identity Processing?

**Figure 1.**
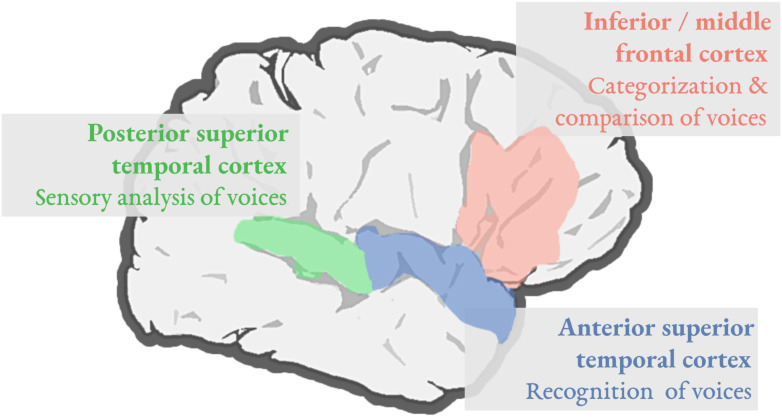
Vocal identity processing is supported by a right-lateralized system involving the posterior superior temporal cortex (green), the anterior superior temporal cortex (blue), and the inferior/middle frontal cortex (pink). The right superior temporal cortex has been implicated in mapping vocal acoustic information to a person’s identity, with posterior regions underlying the early sensory analysis of voices and more anterior regions supporting vocal identity recognition. Left temporal regions (not shown) may contribute to vocal identity processing, with their involvement potentially depending on the familiarity of the voice being processed. Right inferior and middle frontal regions play a role during the categorization of vocal stimuli into task-relevant categories, as well as when listeners must compare a target voice to a vocal sample in working memory, respectively.

A focus on the right hemisphere regions involved in talker processing could inform neurobiological accounts of phonetic processing, at least to the extent that the same right hemisphere regions are recruited for both processes. The association between the right hemisphere and vocal identity processing dates back at least to early clinical studies by Van Lancker and colleagues, who demonstrated that right-hemisphere stroke patients were more likely than left-hemisphere patients to show impairments in identifying the voices of celebrities when performing a forced-choice task (Van Lancker & Canter, 1982; Van Lancker & Kreiman, 1987). Since then, neuroimaging studies have clarified the role of different right hemisphere regions in vocal identity processing (see Maguinness et al., 2018, for a recent review). As illustrated in Figure 1, these studies have revealed that vocal identity processing is largely supported by a set of temporal regions, with posterior temporal regions (shaded green in Figure 1) playing an important role in the early sensory analysis of vocal information, and anterior temporal regions (shaded blue) being important for vocal identity recognition. While not always recruited in vocal identity processing, right frontal brain regions (shaded pink) have been implicated in tasks that require listeners to make comparisons between voices, especially when comparing a vocal sample to a target voice.

#### Temporal lobe contributions to vocal identity processing

Neuroimaging evidence suggests that there is a posterior–anterior gradient in superior temporal lobe responses to vocal information, with right posterior temporal regions being thought to play a larger role in the general sensory processing of voice information (Andics, McQueen, Petersson, et al., 2010; Belin, Zatorre, Lafaille, et al., 2000; Schall et al., 2014; von Kriegstein & Giraud, 2004) and right anterior temporal regions being implicated in mapping from vocal information to a specific identity (Andics, McQueen, Petersson, et al., 2010; Belin, 2006; Belin, Fecteau, & Bédard, 2004; Imaizumi et al., 1997; Nakamura et al., 2001; von Kriegstein & Giraud, 2004). Support for the involvement of posterior superior temporal cortex in vocal identity processing comes from a wide range of studies, including a seminal fMRI study in which Belin, Zatorre, Lafaille, et al. (2000) examined cortical responses when subjects passively listened to human vocal stimuli (both speech sounds and nonspeech vocalizations like laughter) as well as to several types of control stimuli (such as animal sounds, bells, and speech-shaped white noise). Vocal stimuli elicited robust activation in the superior temporal sulcus (STS) bilaterally, but activation on the right was greater both in magnitude and in area than activation on the left. Notably, the response in the right STS was not specific to speech, as activation in the right posterior STS did not differ between speech and nonspeech human vocalizations. Belin, Zatorre, Lafaille, et al. further observed that band-pass filtering the stimuli led to a reduction of STS activation, and this reduction of activation was associated with worsened behavioral performance in a perceptual judgment task conducted outside the scanner (e.g., deciding whether the sounds were vocal or nonvocal). Such results indicate that the right STS is involved in differentiating between vocal and nonvocal auditory information but do not indicate whether it is necessary for such discrimination. Evidence for the latter comes from a study by Bestelmeyer et al. (2011). In that study, the authors first performed a functional localizer to identify the specific parts of right temporal cortex that were recruited when participants passively listened to voices compared to nonvocal auditory stimuli. Subsequent transcranial magnetic stimulation (TMS) to these regions impaired participants’ ability to discriminate between vocal and non-vocal sounds. Taken together, these findings suggest a critical role for the right posterior STS in processing the acoustic detail of human voices.

By contrast, more anterior regions in the right superior temporal cortex seem to be important when listeners need to map these acoustic details to a specific identity. Belin and Zatorre (2003) used fMRI to measure the habituation of neural regions in response to a train of stimuli presented over a short interval. The researchers found that the right anterior STS habituated (i.e., its activity diminished) when listeners encountered a stream of phonologically distinct syllables that were all spoken by the same talker. By contrast, this region did not habituate when listeners encountered a stream of phonologically identical syllables spoken by different talkers. In other words, this region’s response depended on who was producing the speech but not on what the content of the speech was. Convergent evidence comes from Formisano et al. (2008), who collected fMRI data while participants passively listened to different vowels spoken by different talkers. The authors then trained a machine learning algorithm to classify stimuli on the basis of talker identity (ignoring vowel identity) and found that the most discriminative voxels were located in right anterior STS. More recently, Luzzi et al. (2018) reported a case study of a patient who had suffered a stroke that affected his right anterior STS but did not affect posterior temporal regions; while the patient was unimpaired in his ability to indicate whether two voices were the same or different, he was no longer able to recognize his favorite singers on the basis of their voices alone. Overall, these findings suggest a role for right anterior temporal regions in recognizing vocal identity, as opposed to low-level processing of voice information.

Consistent with this view, a number of other studies have found that right anterior temporal regions are recruited when listeners must match vocal details to a known vocal identity. In an fMRI study by von Kriegstein, Eger, et al. (2003), for instance, greater right anterior STS activation was observed when listeners attended to vocal information compared to linguistic information. Similar results were observed in an MEG study by Schall et al. (2014), in which greater right anterior STS activity was observed when subjects had to match a sample of speech to a name compared to when they had to indicate whether a probe word had been present in the speech stream. Moreover, the authors observed a strong correlation between the degree of right anterior STS activity and subjects’ behavioral accuracy on this talker judgment task, suggesting that the variability in the activity of the right anterior STS might underlie individual differences in voice recognition. One way to conceptualize these results is to note that in both the study by von Kriegstein, Eger, et al. (2003) and the study by Schall et al. (2014), listeners were required to compare the incoming auditory signal to their internal representation of a particular vocal identity. As such, the findings indicate that the right anterior STS may play an important role in matching complex auditory objects to a stored vocal representation.

The suggestion that right anterior temporal regions are important for identifying a person on the basis of their voice is particularly striking given studies indicating that the right anterior temporal cortex is vital for person recognition more broadly (Gainotti, 2007). Individuals with damage to the right anterior temporal lobe may show selective impairments in identifying people on the basis of their faces (Damasio, 1990; Gainotti et al., 2003; Tranel et al., 1997) or voices (Gainotti et al., 2003) alone. As such, right temporal regions are thought to be critically involved in integrating perceptual information with conceptual person-specific knowledge (Gainotti, 2007). Consistent with this view, Ross et al. (2010) demonstrated that transcranial direct current stimulation of the right anterior temporal lobe modulated the likelihood that individuals would recover from a tip-of-the-tongue state when naming celebrities from their photographs. However, no such effect of stimulation was observed when subjects were shown photographs of famous places. Such findings point to a critical role of right anterior temporal regions in representing semantic knowledge about person identity specifically. As such, the involvement of right anterior temporal regions in vocal identity recognition may reflect access to multimodal information related to person identity (Maguinness et al., 2018; Perrodin et al., 2015).

While vocal identity processing is supported predominantly by right hemisphere regions, there has been some evidence for left hemisphere involvement in this process. In a study by von Kriegstein and Giraud (2004), for instance, listeners heard speech from talkers who were personally known to them, as well as speech from relatively unfamiliar talkers, to whom listeners’ previous exposure was limited to a few audio clips presented during a familiarization phase. Participants heard several sentences spoken by both the familiar and unfamiliar talkers; on each trial, they had to make a judgment either about the verbal content or about the vocal identity. Making judgments about vocal identity elicited robust activation of both the right posterior and right anterior STS, consistent with the characterization of the right posterior STS being involved in sensory processing of vocal identity and the right anterior STS being involved in vocal identity recognition. The researchers then examined whether functional connectivity with these right temporal regions differed as a function of whether the talkers were personally known to the participants. When participants listened to familiar talkers, there was robust connectivity among different subregions of the right superior temporal lobe. By contrast, when participants heard unfamiliar talkers, there was robust connectivity between the right posterior temporal lobe and the left posterior temporal lobe, suggesting that talker familiarity may modulate the involvement of left hemisphere regions in vocal identity processing. Other studies have supported the notion that the involvement of left temporal cortex in vocal identity processing may differ as a function of talker familiarity (Roswandowitz et al., 2018), and additional work suggests that language familiarity may similarly modulate the involvement of left hemisphere regions in vocal identity processing (Perrachione et al., 2009). Nonetheless, at least one study of stroke patients found that while individuals with right hemisphere damage were impaired in recognizing familiar voices, the performance of patients with left hemisphere damage was comparable to that of healthy controls (Lang et al., 2009); that is, there was no evidence for a left hemisphere role in processing familiar voices. Though additional work is needed to clarify the precise contributions of left temporal cortex, extant data suggest that left posterior temporal regions may play at least some role in vocal identity processing. Nevertheless, the role of the left hemisphere in processing vocal identity information is clearly limited, especially in contrast to the well-established role of the right hemisphere.

#### Frontal lobe contributions to vocal identity processing

In addition to a role for the right temporal lobe, some studies have posited a role for right frontal regions in vocal identity recognition, particularly during tasks that require listeners to categorize voices (Andics, McQueen, & Petersson, 2013; Jones et al., 2015; Zäske et al., 2017) or that require listeners to compare a voice sample to a referent in working memory (Stevens, 2004). Some evidence for the former comes from a study by Andics, McQueen, and Petersson (2013), who presented listeners with a vocal morph continuum where stimuli consisted of two different voices blended in different proportions. Training was used to establish a category boundary between the two voices, and participants then completed an fMRI session in which they had to categorize steps along the morph continuum. Subsequently, a second set of training sessions was administered to establish a new category boundary, after which participants completed a second fMRI session. The authors found that the activation of the right inferior frontal cortex depended on the proximity of a stimulus to the category boundary established during training (regardless of the precise acoustic details). These findings were interpreted as evidence that the right inferior frontal cortex supports the categorization of vocal stimuli into vocal identity categories, with the harder-to-categorize near-boundary stimuli eliciting more activation in right inferior frontal cortex. Consistent with this finding, Jones et al. (2015) observed that stroke patients who had damage to right frontal cortex were impaired in their categorization of talker gender when presented with stimuli from male–female continua; critically, the right STS was intact in these patients, suggesting that these results were not attributable to impairments in early sensory processing. Thus, the right inferior frontal cortex appears to play a critical role in allowing listeners to evaluate voices with respect to known vocal categories, whether these categories are task-relevant (e.g., ones established through training) or socio-indexically derived (i.e., categories based on talker-relevant social cues, such as gender or sexual orientation; Johnson, 2008; Munson, 2007).

The right frontal cortex has also been implicated in tasks that require listeners to compare one vocal sample to a second sample held in working memory. In an fMRI study, Stevens (2004) had participants listen to a series of stimuli while performing a two-back working memory task. On some blocks, they had to indicate whether the talker producing the current stimulus was the same as the talker who had produced the stimulus two items previously, and on other blocks, they had to indicate whether the same word had been produced two items previously. Subjects showed greater activation in the right middle frontal gyrus when performing the talker two-back task and greater activation in left inferior frontal gyrus when performing the word two-back task. Such a finding suggests a role for right frontal regions when subjects have to make explicit comparisons about vocal identity across stimuli.

Strikingly, the role of right frontal brain areas in vocal identity recognition seems to parallel a similar role for left frontal regions in phonological processing during speech perception. Just as the right inferior frontal cortex is strongly recruited when listeners hear stimuli near a vocal category boundary, the left inferior frontal cortex has been shown to be robustly activated by stimuli near a phonetic category boundary (Myers, 2007). Similarly, right frontal regions are recruited when demands on vocal working memory are high, just as left frontal regions are recruited when demands on phonological processing are high (Burton et al., 2000). More generally, the extant literature suggests that vocal identity processing is supported by a right-lateralized neural system, whereas speech perception is supported by an analogous left-lateralized system. To the extent that phonetic processing is influenced by talker information (as described in How Is Phonetic Processing Linked to Talker Processing?), it is worth considering how the right hemisphere may interact with the left to support speech perception; I turn to this question next.

### How Might the Right Hemisphere Support Speech Perception?

Though the leftward lateralization of language processing represents a core feature of current neurobiological models of speech perception (Binder, Frost, et al., 1997; Binder, Swanson, et al., 1996; Geschwind, 1970; Hickok & Poeppel, 2000, 2004, 2007; Rauschecker & Scott, 2009), there is nevertheless some evidence that the right hemisphere—and right temporal cortex in particular—does play a role in speech perception. At least one study (Boatman et al., 1998) demonstrated intact syllable discrimination in a patient whose left hemisphere was sedated through a sodium amobarbital injection (Wada & Rasmussen, 1960), and functional neuroimaging studies of speech perception routinely implicate right temporal structures in speech perception (Belin, Zatorre, Hoge, et al., 1999; Blumstein et al., 2005; Davis et al., 2011; Giraud et al., 2004; Turkeltaub & Branch Coslett, 2010; Zatorre et al., 1996). More recently, a study by Kennedy-Higgins et al. (2020) found that listeners’ ability to repeat speech presented against background noise was impaired when they received TMS above either the left or right superior temporal gyrus (STG), but not when stimulation was performed at a control site. Collectively, such findings suggest a nonnegligible role for the right hemisphere in speech perception.

However, while left and right temporal structures are both routinely recruited for speech perception, they do not respond equally to acoustic information. In particular, left temporal regions seem to respond preferentially to rapid changes in the auditory signal, whereas right temporal regions appear to have a general preference for processing low-frequency modulations in the acoustic signal (Belin, McAdams, et al., 1998; Robin et al., 1990; Schwartz & Tallal, 1980; Scott et al., 2000). On the basis of these and other findings, Poeppel (2003) proposed the asymmetric sampling in time (AST) hypothesis. Under this view, the left hemisphere samples the speech signal at a relatively fast rate (40 Hz) and as such is well-suited for processing rapidly changing acoustic information (fluctuations on the order of approximately 25 ms); as such, left temporal processing is thought to be reflected in neuronal oscillations that occur in the gamma frequency band. By contrast, the right hemisphere has a slower rate of temporal integration (5 Hz), allowing it to process signal fluctuations that occur on the order of approximately 200 ms; right temporal activity is thought to be reflected in theta-band neuronal oscillations. Notably, the right hemisphere preference for low-frequency modulations has been observed both with speech (Abrams et al., 2008) and nonspeech stimuli (Boemio et al., 2005; Zatorre & Belin, 2001), suggesting that asymmetric sampling is a core property of temporal cortex rather than being specific to speech perception. Key to the AST hypothesis is the premise that the processing preferences of the two hemispheres depend on the physical properties of the auditory signal.

The AST can readily explain an association between the right hemisphere and processing the prosody of speech, for instance, as prosodic cues are conveyed over a relatively large temporal window (Poeppel, 2003). However, rightward lateralization is not always observed for prosodic processing, with the precise lateralization depending on a number of factors, including the control task used (Kreitewolf, Friederici, & von Kriegstein, 2014). Moreover, a number of studies have demonstrated left hemisphere involvement in prosodic processing when such information conveys linguistic information, whether lexical (Gandour, Tong, et al., 2004; Gandour, Wong, et al., 2002) or syntactic (van der Burght et al., 2019). In one such study, van der Burght et al. observed robust activation of the left inferior frontal gyrus when prosodic information in a speech sample determined syntactic structure but not when prosody was not needed for resolving the sentence’s syntax. These results are consistent with the view that while hemispheric asymmetries in processing auditory information may be partly attributable to the physical acoustic properties of the signal, the extent to which each hemisphere is involved may also largely depend on the functional use of the signal (Van Lancker, 1980).

The functional view predicts that right hemisphere involvement in speech perception is not limited simply to instances when listeners integrate auditory information over a long temporal window—rather, *the involvement of the right hemisphere in speech perception may specifically reflect the process of conditioning phonetic processing on talker information* (Kreitewolf, Gaudrain, & von Kriegstein, 2014b; Luthra, Correia, et al., 2020; Myers & Mesite, 2014; Myers & Theodore, 2017; von Kriegstein, Smith, et al., 2010). Some evidence for this hypothesis comes from a study by von Kriegstein, Smith, et al. (2010), in which listeners heard stimulus trains that varied in syllable identity, amplitude, and/or vocal tract length (an acoustic parameter that differs across talkers). Listeners performed either a one-back speech task (in which they had to indicate if the current stimulus matched the preceding stimulus in syllable identity) or a control task (either a one-back talker task or a one-back amplitude task). The authors observed that the left posterior STG was sensitive to vocal tract length (i.e., to acoustic information associated with talker identity). Moreover, von Kriegstein, Smith, et al. (2010) found that during the speech task, the functional connections between the left posterior STG and its right hemisphere analogue differed as a function of vocal tract length. The authors interpreted their findings as evidence that when listeners process talker-specific information in support of speech recognition, both the left and right temporal cortex are recruited.

Additional support for this perspective comes from a study by Myers and Theodore (2017), in which listeners were exposed to two talkers who differed in their productions of the sound /k/. Specifically, the talkers differed in whether they produced /k/ with a relatively short or long VOT (an acoustic-phonetic cue that distinguishes the voiceless sound /k/ from its voiced counterpart, /g/); notably, processing VOT requires integrating over a relatively short temporal window. After being familiarized with these two talkers, listeners completed an MRI scan during which they performed phonetic categorization on the words “cane” and “gain”; critically, during this phonetic categorization task, listeners heard both talker-typical and talker-atypical variants of the word “cane.” Myers and Theodore found that the functional activation of the right STG depended on whether the “cane” variant heard was typical or atypical of that talker. Such a result is consistent with the functional view of hemispheric asymmetries, which holds that despite being a short-duration cue, VOT would be processed by the right hemisphere if it was informative of talker identity. Additionally, the authors observed that the more typical the acoustic-phonetic variant was of a talker, the more tightly coupled the activity between the right STG and left temporal cortex. Taken together, these findings support the perspective that the right temporal cortex may support a listener’s ability to adapt to the idiosyncratic ways that different talkers produce their speech sounds; this may be achieved through the activity of the right temporal cortex itself or through interactions between the right temporal cortex and left temporal regions associated with phonetic processing.

**Figure 2.**
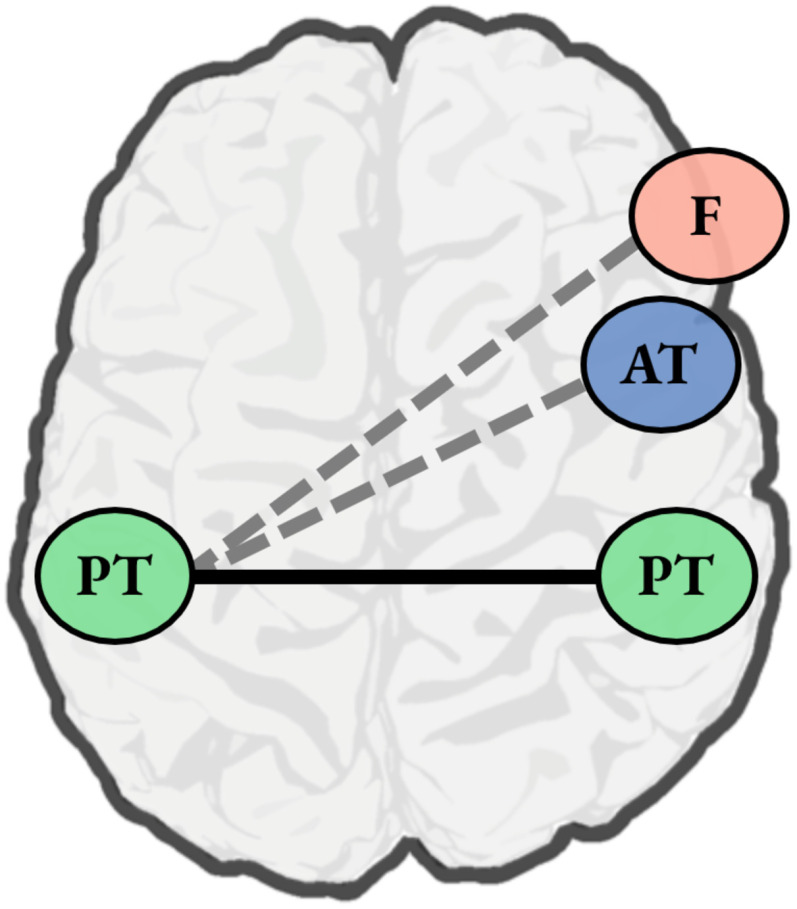
Speech perception involves interactions between left posterior temporal regions implicated in phonetic processing and right posterior temporal regions associated with the perceptual analysis of vocal information. These interactions may specifically reflect the process of conditioning phonetic identity on talker information. In this figure, posterior temporal regions are depicted by green circles with the label “PT,” and the established functional connection between them is indicated via a solid black line. However, the literature on speech perception does not suggest a strong role for other regions involved in vocal identity processing—namely, right anterior temporal cortex (blue circle labeled “AT”) and right inferior/middle frontal cortex (pink circle labeled “F”). It may be the case that these other right hemisphere regions only interact with left posterior temporal cortex (dashed gray lines) when demands on talker processing are high.

While there are documented functional connections between left posterior temporal regions involved in phonetic processing and right posterior temporal regions involved in the early analysis of vocal detail, there does not appear to be a strong role for functional connections between left posterior temporal regions and other right hemisphere regions associated with vocal identity processing (Figure 2). In considering why this might be, it is worth noting that these other regions are primarily associated with explicitly mapping vocal information to a known identity (in the case of right anterior temporal areas) or are recruited only when listeners are tasked with categorizing or comparing between vocal samples (in the case of right frontal regions). That is, these regions are only recruited when demands on vocal identity processing are high.

In ecological instances of speech perception, however, listeners may not need to make explicit judgments about talker identity; indeed, listeners can typically leverage myriad sources of context to identify a talker’s intended phoneme, be they syntactic (Fox & Blumstein, 2016), semantic (Borsky et al., 1998), lexical (Ganong, 1980), or visual (Frost et al., 1988; McGurk & MacDonald, 1976). As such, the involvement of right anterior temporal and right frontal regions in phonetic processing may be limited to situations where the demands on the talker identification system is high, such that talker identity uniquely determines the mapping between acoustics and phonemes. I suggest that future studies assess this hypothesis directly, investigating both the activation of these right hemisphere regions and their functional connections to left temporal regions involved in phonetic processing.

Furthermore, the observation that naturalistic speech perception does not necessarily place a strong burden on talker processing systems may hint at why frank deficits in speech perception are not observed in individuals with right hemisphere damage. I suggest that the impact of right hemisphere damage (and damage to right posterior temporal cortex in particular) may only be observable in tasks that specifically require listeners to condition phonetic identity on talker information. Future work testing this hypothesis in right hemisphere patients will therefore be important in elucidating a potential subclinical impairment.

## DISCUSSION

The acoustic signal simultaneously conveys linguistic information about speech sounds as well as nonlinguistic information about vocal identity, and in general, the process of speech perception is not independent from processing talker information (Mullennix & Pisoni, 1990). In this review, I have attempted to clarify the nature of right hemisphere involvement in speech perception by focusing on its role in vocal identity processing. As depicted in Figure 1, vocal identity processing entails the contributions of right posterior temporal cortex, right anterior temporal cortex, and right inferior/middle frontal cortex. Based on the functional view of hemispheric contributions to processing auditory information (Van Lancker, 1980), I presented evidence that the recruitment of right posterior temporal regions during speech perception may reflect the process of conditioning phonetic identity on talker information. I noted that right anterior temporal and right frontal regions are not strongly implicated during speech perception (Figure 2), and I suggested that the limited involvement of these regions may reflect the fact that in ecological speech perception, demands on talker processing are relatively low. In closing, I suggest that our understanding of the role of the right hemisphere in speech perception may be improved by focusing specifically on conditions where demands on talker processing are high (e.g., when a listener must appeal to talker information in order to know how to map the speech signal onto phonetic categories). Future work of this sort may also elucidate potential subclinical impairments in speech perception in individuals who have sustained damage to the right hemisphere.

## ACKNOWLEDGMENTS

I am thankful to Emily Myers, Jim Magnuson, Rachel Theodore, Gerry Altmann, Eiling Yee, Jonathan Peelle, and three anonymous reviewers for their feedback on previous versions of this manuscript. This work was supported by an NSF Graduate Research Fellowship awarded to the author. The publication of this work was supported by the program in Science of Learning & Art of Communication at the University of Connecticut, which is supported by the National Science Foundation under Grant DGE-1747486.

## FUNDING INFORMATION

Sahil Luthra, National Science Foundation (http://dx.doi.org/10.13039/501100008982), Award ID: Graduate Research Fellowship. James S. Magnuson, National Science Foundation (http://dx.doi.org/10.13039/501100008982), Award ID: NRT 1747486.

## References

[bib1] Abrams, D. A. , Nicol, T. , Zecker, S. , & Kraus, N. (2008). Right-hemisphere auditory cortex is dominant for coding syllable patterns in speech. The Journal of Neuroscience, 28(15), 3958–3965. DOI: https://doi.org/10.1523/JNEUROSCI.0187-08.2008, PMID: 18400895, PMCID: PMC2713056 18400895 10.1523/JNEUROSCI.0187-08.2008 PMC2713056

[bib2] Allen, J. S. , & Miller, J. L. (2004). Listener sensitivity to individual talker differences in voice-onset-time. The Journal of the Acoustical Society of America, 115(6), 3171–3183. DOI: https://doi.org/10.1121/1.1701898, PMID: 15237841 15237841 10.1121/1.1701898

[bib3] Allen, J. S. , Miller, J. L. , & DeSteno, D. (2003). Individual talker differences in voice-onset-time. The Journal of the Acoustical Society of America, 113(1), 544–552. DOI: https://doi.org/10.1121/1.1528172, PMID: 12558290 12558290 10.1121/1.1528172

[bib4] Andics, A. , McQueen, J. M. , & Petersson, K. M. (2013). Mean-based neural coding of voices. NeuroImage, 79, 351–360. DOI: https://doi.org/10.1016/j.neuroimage.2013.05.002, PMID: 23664949 23664949 10.1016/j.neuroimage.2013.05.002

[bib5] Andics, A. , McQueen, J. M. , Petersson, K. M. , Gál, V. , Rudas, G. , & Vidnyánszky, Z. (2010). Neural mechanisms for voice recognition. NeuroImage, 52(4), 1528–1540. DOI: https://doi.org/10.1016/j.neuroimage.2010.05.048, PMID: 20553895 20553895 10.1016/j.neuroimage.2010.05.048

[bib6] Belin, P. (2006). Voice processing in human and non-human primates. Philosophical Transactions of the Royal Society B: Biological Sciences, 361(1476), 2091–2107. DOI: https://doi.org/10.1098/rstb.2006.1933, PMID: 17118926, PMCID: PMC1764839 10.1098/rstb.2006.1933 PMC1764839 17118926

[bib7] Belin, P. , Fecteau, S. , & Bédard, C. (2004). Thinking the voice: Neural correlates of voice perception. Trends in Cognitive Sciences, 8(3), 129–135. DOI: https://doi.org/10.1016/j.tics.2004.01.008, PMID: 15301753 15301753 10.1016/j.tics.2004.01.008

[bib8] Belin, P. , McAdams, S. , Smith, B. , Savel, S. , Thivard, L. , Samson, S. , & Samson, Y. (1998). The functional anatomy of sound intensity discrimination. Journal of Neuroscience, 18(16), 6388–6394. DOI: https://doi.org/10.1523/JNEUROSCI.18-16-06388.1998, PMID: 9698330, PMCID: PMC6793181 9698330 10.1523/JNEUROSCI.18-16-06388.1998 PMC6793181

[bib9] Belin, P. , & Zatorre, R. J. (2003). Adaptation to speaker’s voice in right anterior temporal lobe. Neuroreport, 14(16), 2105–2109. DOI: https://doi.org/10.1097/00001756-200311140-00019, PMID: 14600506 14600506 10.1097/00001756-200311140-00019

[bib10] Belin, P. , Zatorre, R. J. , Hoge, R. , Evans, A. C. , & Pike, B. (1999). Event-related fMRI of the auditory cortex. NeuroImage, 10(4), 417–429. DOI: https://doi.org/10.1006/nimg.1999.0480, PMID: 10493900 10493900 10.1006/nimg.1999.0480

[bib11] Belin, P. , Zatorre, R. J. , Lafaille, P. , Ahad, P. , & Pike, B. (2000). Voice-selective areas in human auditory cortex. Nature, 403(6767), 309–312. DOI: https://doi.org/10.1038/35002078, PMID: 10659849 10659849 10.1038/35002078

[bib12] Bestelmeyer, P. E. G. , Belin, P. , & Grosbras, M.-H. (2011). Right temporal TMS impairs voice detection. Current Biology, 21(20), R838–R839. DOI: https://doi.org/10.1016/j.cub.2011.08.046, PMID: 22032183 22032183 10.1016/j.cub.2011.08.046

[bib13] Binder, J. R. , Frost, J. A. , Hammeke, T. A. , Cox, R. W. , Rao, S. M. , & Prieto, T. (1997). Human brain language areas identified by functional magnetic resonance imaging. The Journal of Neuroscience, 17(1), 353–362. DOI: https://doi.org/10.1523/JNEUROSCI.17-01-00353.1997, PMID: 8987760, PMCID: PMC6793702 8987760 10.1523/JNEUROSCI.17-01-00353.1997 PMC6793702

[bib14] Binder, J. R. , Swanson, S. J. , Hammeke, T. A. , Morris, G. L. , Mueller, W. M. , Fischer, M. , Benbadis, S. , Frost, J. A. , Rao, S. M. , & Haughton, V. M. (1996). Determination of language dominance using functional MRI. Neurology, 46, 978–984. DOI: https://doi.org/10.1212/WNL.46.4.978, PMID: 8780076 8780076 10.1212/wnl.46.4.978

[bib15] Blumstein, S. E. , & Myers, E. B. (2014). Neural systems underlying speech perception. In K. N. Ochsner & S. Kosslyn (Eds.), The Oxford Handbook of Cognitive Neuroscience, Volume 1 (pp. 507–523). Oxford University Press. DOI: 10.1093/oxfordhb/9780199988693.013.0025

[bib16] Blumstein, S. E. , Myers, E. B. , & Rissman, J. (2005). The perception of voice onset time: An fMRI investigation of phonetic category structure. Journal of Cognitive Neuroscience, 17(9), 1353–1366. DOI: https://doi.org/10.1162/0898929054985473, PMID: 16197689 16197689 10.1162/0898929054985473

[bib17] Boatman, D. , Hart, J. , Lesser, R. P. , Honeycutt, N. , Anderson, N. B. , Miglioretti, D. , & Gordon, B. (1998). Right hemisphere speech perception revealed by amobarbital injection and electrical interference. Neurology, 51(2), 458–464. DOI: https://doi.org/10.1212/WNL.51.2.458, PMID: 9710019 9710019 10.1212/wnl.51.2.458

[bib18] Boemio, A. , Fromm, S. , Braun, A. , & Poeppel, D. (2005). Hierarchical and asymmetric temporal sensitivity in human auditory cortices. Nature Neuroscience, 8(3), 389–395. DOI: https://doi.org/10.1038/nn1409, PMID: 15723061 15723061 10.1038/nn1409

[bib19] Borsky, S. , Tuller, B. , & Shapiro, L. P. (1998). “How to milk a coat:” The effects of semantic and acoustic information on phoneme categorization. The Journal of the Acoustical Society of America, 103(5), 2670–2676. DOI: https://doi.org/10.1121/1.422787, PMID: 9604360 9604360 10.1121/1.422787

[bib20] Broca, P. (1861). Remarques sur le siège de la faculté du langage articulé, suivies d’une observation d’aphémie (perte de la parole). Bulletin et Memoires de La Société Anatomique de Paris, 6, 330–357.

[bib21] Burton, M. W. , Small, S. L. , & Blumstein, S. E. (2000). The role of segmentation in phonological processing: An fMRI investigation. Journal of Cognitive Neuroscience, 12(4), 679–690. DOI: https://doi.org/10.1162/089892900562309, PMID: 10936919 10936919 10.1162/089892900562309

[bib22] Clayards, M. , Tanenhaus, M. K. , Aslin, R. N. , & Jacobs, R. A. (2008). Perception of speech reflects optimal use of probabilistic speech cues. Cognition, 108(3), 804–809. DOI: https://doi.org/10.1016/j.cognition.2008.04.004, PMID: 18582855, PMCID: PMC2582186 18582855 10.1016/j.cognition.2008.04.004 PMC2582186

[bib23] Damasio, A . (1990). Face agnosia and the neural substrates of memory. Annual Review of Neuroscience, 13(1), 89–109. DOI: https://doi.org/10.1146/annurev.ne.13.030190.000513, PMID: 2183687 10.1146/annurev.ne.13.030190.000513 2183687

[bib24] Davis, M. H. , Ford, M. A. , Kherif, F. , & Johnsrude, I. S. (2011). Does semantic context benefit speech understanding through “top–down” processes? Evidence from time-resolved sparse fMRI. Journal of Cognitive Neuroscience, 23(12), 3914–3932. DOI: https://doi.org/10.1162/jocn_a_00084, PMID: 21745006 21745006 10.1162/jocn_a_00084

[bib25] Desai, R. , Liebenthal, E. , Waldron, E. , & Binder, J. R. (2008). Left posterior temporal regions are sensitive to auditory categorization. Journal of Cognitive Neuroscience, 20(7), 1174–1188. DOI: https://doi.org/10.1162/jocn.2008.20081, PMID: 18284339, PMCID: PMC3350814 18284339 10.1162/jocn.2008.20081 PMC3350814

[bib26] Formisano, E. , De Martino, F. , Bonte, M. , & Goebel, R. (2008). “Who” is saying “what”? Brain-based decoding of human voice and speech. Science, 322(5903), 970–973. DOI: https://doi.org/10.1126/science.1164318, PMID: 18988858 18988858 10.1126/science.1164318

[bib27] Fox, N. P. , & Blumstein, S. E. (2016). Top-down effects of syntactic sentential context on phonetic processing. Journal of Experimental Psychology: Human Perception and Performance, 42(5), 730–741. DOI: https://doi.org/10.1037/a0039965, PMID: 26689310 26689310 10.1037/a0039965

[bib28] Frost, R. , Repp, B. H. , & Katz, L. (1988). Can speech perception be influenced by simultaneous presentation of print? Journal of Memory and Language, 27(6), 741–755. DOI: 10.1016/0749-596X(88)90018-6

[bib29] Gainotti, G. (2007). Different patterns of famous people recognition disorders in patients with right and left anterior temporal lesions: A systematic review. Neuropsychologia, 45(8), 1591–1607. DOI: https://doi.org/10.1016/j.neuropsychologia.2006.12.013, PMID: 17275042 17275042 10.1016/j.neuropsychologia.2006.12.013

[bib30] Gainotti, G. , Barbier, A. , & Marra, C. (2003). Slowly progressive defect in recognition of familiar people in a patient with right anterior temporal atrophy. Brain, 126(4), 792–803. DOI: https://doi.org/10.1093/brain/awg092, PMID: 12615639 12615639 10.1093/brain/awg092

[bib31] Gandour, J. , Tong, Y. , Wong, D. , Talavage, T. , Dzemidzic, M. , Xu, Y. , Li, X. , & Lowe, M. (2004). Hemispheric roles in the perception of speech prosody. NeuroImage, 23(1), 344–357. DOI: https://doi.org/10.1016/j.neuroimage.2004.06.004, PMID: 15325382 15325382 10.1016/j.neuroimage.2004.06.004

[bib32] Gandour, J. , Wong, D. , Lowe, M. , Dzemidzic, M. , Satthamnuwong, N. , Tong, Y. , & Li, X. (2002). A cross-linguistic fMRI study of spectral and temporal cues underlying phonological processing. Journal of Cognitive Neuroscience, 14(7), 1076–1087. DOI: https://doi.org/10.1162/089892902320474526, PMID: 12419130 12419130 10.1162/089892902320474526

[bib33] Ganong, W. F. (1980). Phonetic categorization in auditory word perception. Journal of Experimental Psychology: Human Perception and Performance, 6(1), 110–125. DOI: 10.1037/0096-1523.6.1.110 6444985

[bib34] Geschwind, N. (1970). The organization of language and the brain. Science, 170(3961), 940–944. DOI: https://doi.org/10.1126/science.170.3961.940, PMID: 5475022 5475022 10.1126/science.170.3961.940

[bib35] Giraud, A. L. , Kell, C. , Thierfelder, C. , Sterzer, P. , Russ, M. O. , Preibisch, C. , & Kleinschmidt, A. (2004). Contributions of sensory input, auditory search and verbal comprehension to cortical activity during speech processing. Cerebral Cortex, 14(3), 247–255. DOI: https://doi.org/10.1093/cercor/bhg124, PMID: 14754865 14754865 10.1093/cercor/bhg124

[bib36] Goggin, J. P. , Thompson, C. P. , Strube, G. , & Simental, L. R. (1991). The role of language familiarity in voice identification. Memory & Cognition, 19(5), 448–458. DOI: https://doi.org/10.3758/BF03199567, PMID: 1956306 1956306 10.3758/bf03199567

[bib37] Heilman, K. M. , Bowers, D. , Speedie, L. , & Branch Coslett, H. (1984). Comprehension of affective and nonaffective prosody. Neurology, 34(7), 917–921. DOI: https://doi.org/10.1212/WNL.34.7.917, PMID: 6539867 6539867 10.1212/wnl.34.7.917

[bib38] Hickok, G. , & Poeppel, D. (2000). Towards a functional neuroanatomy of speech perception. Trends in Cognitive Sciences, 4(4), 131–138. DOI: 10.1016/S1364-6613(00)01463-7 10740277

[bib39] Hickok, G. , & Poeppel, D. (2004). Dorsal and ventral streams: A framework for understanding aspects of the functional anatomy of language. Cognition, 92(1–2), 67–99. DOI: https://doi.org/10.1016/j.cognition.2003.10.011, PMID: 15037127 15037127 10.1016/j.cognition.2003.10.011

[bib40] Hickok, G. , & Poeppel, D. (2007). The cortical organization of speech processing. Nature Reviews Neuroscience, 8(5), 393–402. DOI: https://doi.org/10.1038/nrn2113, PMID: 17431404 17431404 10.1038/nrn2113

[bib41] Holmes, E. , Domingo, Y. , & Johnsrude, I. S. (2018). Familiar voices are more intelligible, even if they are not recognized as familiar. Psychological Science, 29(10), 1575–1583. DOI: https://doi.org/10.1177/0956797618779083, PMID: 30096018 30096018 10.1177/0956797618779083

[bib42] Holmes, E. , & Johnsrude, I. S. (2020). Speech spoken by familiar people is more resistant to interference by linguistically similar speech. Journal of Experimental Psychology: Learning, Memory and Cognition, 46(8), 1465–1476. DOI: https://doi.org/10.1037/xlm0000823, PMID: 32105143 32105143 10.1037/xlm0000823

[bib43] Imaizumi, S. , Mori, K. , Kiritani, S. , Kawashima, R. , Sugiura, M. , Fukuda, H. , Itoh, K. , Kato, T. , Nakamura, A. , Hatano, K. , Kojima, S. , & Nakamura, K. (1997). Vocal identification of speaker and emotion activates differerent brain regions. NeuroReport, 8(12), 2809–2812. DOI: https://doi.org/10.1097/00001756-199708180-00031, PMID: 9295122 9295122 10.1097/00001756-199708180-00031

[bib44] Johnson, K. A. (2008). Speaker normalization in speech perception. In D. B. Pisoni & R. E. Remez (Eds.), The handbook of speech perception (pp. 363–389). Blackwell Publishing. DOI: 10.1002/9780470757024.ch15

[bib45] Johnsrude, I. S. , Mackey, A. , Hakyemez, H. , Alexander, E. , Trang, H. P. , & Carlyon, R. P. (2013). Swinging at a cocktail party: Voice familiarity aids speech perception in the presence of a competing voice. Psychological Science, 24(10), 1995–2004. DOI: https://doi.org/10.1177/0956797613482467, PMID: 23985575 23985575 10.1177/0956797613482467

[bib46] Jones, A. B. , Farrall, A. J. , Belin, P. , & Pernet, C. R. (2015). Hemispheric association and dissociation of voice and speech information processing in stroke. Cortex, 71, 232–239. DOI: https://doi.org/10.1016/j.cortex.2015.07.004, PMID: 26247409 26247409 10.1016/j.cortex.2015.07.004

[bib47] Joos, M . (1948). Acoustic phonetics. Language, 24(2), 5–136. DOI: 10.2307/522229

[bib48] Kennedy-Higgins, D. , Devlin, J. T. , Nuttall, H. E. , & Adank, P. (2020). The causal role of left and right superior temporal gyri in speech perception in noise: A transcranial magnetic stimulation study. Journal of Cognitive Neuroscience, 32(6), 1092–1103. DOI: https://doi.org/10.1162/jocn_a_01521, PMID: 31933438 31933438 10.1162/jocn_a_01521

[bib49] Kleinschmidt, D. F. (2019). Structure in talker variability: How much is there and how much can it help? Language, Cognition and Neuroscience, 34(1), 43–68. DOI: https://doi.org/10.1080/23273798.2018.1500698, PMID: 30619905, PMCID: PMC6320234 10.1080/23273798.2018.1500698 PMC6320234 30619905

[bib50] Kleinschmidt, D. F. , & Jaeger, T. F. (2015). Robust speech perception: Recognize the familiar, generalize to the similar, and adapt to the novel. Psychological Review, 122(2), 148–203. DOI: https://doi.org/10.1037/a0038695, PMID: 25844873, PMCID: PMC4744792 25844873 10.1037/a0038695 PMC4744792

[bib51] Kraljic, T. , & Samuel, A. G. (2005). Perceptual learning for speech: Is there a return to normal? Cognitive Psychology, 51(2), 141–178. DOI: https://doi.org/10.1016/j.cogpsych.2005.05.001, PMID: 16095588 16095588 10.1016/j.cogpsych.2005.05.001

[bib52] Kreitewolf, J. , Friederici, A. D. , & von Kriegstein, K. (2014). Hemispheric lateralization of linguistic prosody recognition in comparison to speech and speaker recognition. NeuroImage, 102(P2), 332–344. DOI: https://doi.org/10.1016/j.neuroimage.2014.07.038, PMID: 25087482 25087482 10.1016/j.neuroimage.2014.07.038

[bib53] Kreitewolf, J. , Gaudrain, E. , & von Kriegstein, K. (2014). A neural mechanism for recognizing speech spoken by different speakers. NeuroImage, 91, 375–385. DOI: https://doi.org/10.1016/j.neuroimage.2014.01.005, PMID: 24434677 24434677 10.1016/j.neuroimage.2014.01.005

[bib54] Kreitewolf, J. , Mathias, S. R. , & von Kriegstein, K. (2017). Implicit talker training improves comprehension of auditory speech in noise. Frontiers in Psychology, 8(SEP), 1–8. DOI: https://doi.org/10.3389/fpsyg.2017.01584, PMID: 28959226, PMCID: PMC5603660 28959226 10.3389/fpsyg.2017.01584 PMC5603660

[bib55] Lang, C. J. G. , Kneidl, O. , Hielscher-Fastabend, M. , & Heckmann, J. G. (2009). Voice recognition in aphasic and non-aphasic stroke patients. Journal of Neurology, 256(8), 1303–1306. DOI: https://doi.org/10.1007/s00415-009-5118-2, PMID: 19353219 19353219 10.1007/s00415-009-5118-2

[bib56] Lee, Y.-S. , Turkeltaub, P. , Granger, R. , & Raizada, R. D. S. (2012). Categorical speech processing in Broca’s area: An fMRI study using multivariate pattern-based analysis. Journal of Neuroscience, 32(11), 3942–3948. DOI: https://doi.org/10.1523/JNEUROSCI.3814-11.2012, PMID: 22423114, PMCID: PMC6703443 22423114 10.1523/JNEUROSCI.3814-11.2012 PMC6703443

[bib57] Liebenthal, E. , Desai, R. , Ellingson, M. M. , Ramachandran, B. , Desai, A. , & Binder, J. R. (2010). Specialization along the left superior temporal sulcus for auditory categorization. Cerebral Cortex, 20(12), 2958–2970. DOI: https://doi.org/10.1093/cercor/bhq045, PMID: 20382643, PMCID: PMC2978244 20382643 10.1093/cercor/bhq045 PMC2978244

[bib58] Luthra, S. , Correia, J. M. , Kleinschmidt, D. F. , Mesite, L. M. , & Myers, E. B. (2020). Lexical information guides retuning of neural patterns in perceptual learning for speech. Journal of Cognitive Neuroscience, 32(10), 2001–2012. DOI: https://doi.org/10.1162/jocn_a_01612, PMID: 32662731 32662731 10.1162/jocn_a_01612 PMC8048099

[bib59] Luthra, S. , Guediche, S. , Blumstein, S. E. , & Myers, E. B. (2019). Neural substrates of subphonemic variation and lexical competition in spoken word recognition. Language, Cognition and Neuroscience, 34(2), 141–169. DOI: https://doi.org/10.1080/23273798.2018.1531140, PMID: 31106225, PMCID: PMC6516505 10.1080/23273798.2018.1531140 PMC6516505 31106225

[bib60] Luzzi, S. , Coccia, M. , Polonara, G. , Reverberi, C. , Ceravolo, G. , Silvestrini, M. , Fringuelli, F. , Baldinelli, S. , Provinciali, L. , & Gainotti, G. (2018). Selective associative phonagnosia after right anterior temporal stroke. Neuropsychologia, 116, 154–161. DOI: https://doi.org/10.1016/j.neuropsychologia.2017.05.016, PMID: 28506806 28506806 10.1016/j.neuropsychologia.2017.05.016

[bib61] Maguinness, C. , Roswandowitz, C. , & von Kriegstein, K. (2018). Understanding the mechanisms of familiar voice-identity recognition in the human brain. Neuropsychologia, 116, 179–193. DOI: https://doi.org/10.1016/j.neuropsychologia.2018.03.039, PMID: 29614253 29614253 10.1016/j.neuropsychologia.2018.03.039

[bib62] McGurk, H. , & MacDonald, J. (1976). Hearing lips and seeing voices. Nature, 264, 746–748. DOI: https://doi.org/10.1038/264746a0, PMID: 1012311 1012311 10.1038/264746a0

[bib63] Mesgarani, N. , Cheung, C. , Johnson, K. A. , & Chang, E. F. (2014). Phonetic feature encoding in human superior temporal gyrus. Science, 343(6174), 1006–1011. DOI: https://doi.org/10.1126/science.1245994, PMID: 24482117, PMCID: PMC4350233 24482117 10.1126/science.1245994 PMC4350233

[bib64] Mitchell, R. L. C. , & Crow, T. J. (2005). Right hemisphere language functions and schizophrenia: The forgotten hemisphere? Brain, 128(5), 963–978. DOI: https://doi.org/10.1093/brain/awh466, PMID: 15743870 15743870 10.1093/brain/awh466

[bib65] Mullennix, J. W. , & Pisoni, D. B. (1990). Stimulus variability and processing dependencies in speech perception. Perception & Psychophysics, 47(4), 379–390. DOI: https://doi.org/10.3758/BF03210878, PMID: 2345691, PMCID: PMC3512111 2345691 10.3758/bf03210878 PMC3512111

[bib66] Munson, B. (2007). The acoustic correlates of perceived masculinity, perceived femininity, and perceived sexual orientation. Language and Speech, 50(1), 125–142. DOI: https://doi.org/10.1177/00238309070500010601, PMID: 17518106 17518106 10.1177/00238309070500010601

[bib67] Myers, E. B. (2007). Dissociable effects of phonetic competition and category typicality in a phonetic categorization task: An fMRI investigation. Neuropsychologia, 45(7), 1463–1473. DOI: https://doi.org/10.1016/j.neuropsychologia.2006.11.005, PMID: 17178420, PMCID: PMC1876725 17178420 10.1016/j.neuropsychologia.2006.11.005 PMC1876725

[bib68] Myers, E. B. , Blumstein, S. E. , Walsh, E. , & Eliassen, J. (2009). Inferior frontal regions underlie the perception of phonetic category invariance. Psychological Science, 20(7), 895–903. DOI: https://doi.org/10.1111/j.1467-9280.2009.02380.x, PMID: 19515116, PMCID: PMC2851201 19515116 10.1111/j.1467-9280.2009.02380.x PMC2851201

[bib69] Myers, E. B. , & Mesite, L. M. (2014). Neural systems underlying perceptual adjustment to non-standard speech tokens. Journal of Memory and Language, 76, 80–93. DOI: https://doi.org/10.1016/j.jml.2014.06.007, PMID: 25092949, PMCID: PMC4118215 25092949 10.1016/j.jml.2014.06.007 PMC4118215

[bib70] Myers, E. B. , & Theodore, R. M. (2017). Voice-sensitive brain networks encode talker-specific phonetic detail. Brain and Language, 165, 33–44. DOI: https://doi.org/10.1016/j.bandl.2016.11.001, PMID: 27898342, PMCID: PMC5237402 27898342 10.1016/j.bandl.2016.11.001 PMC5237402

[bib71] Nakamura, K. , Kawashima, R. , Sugiura, M. , Kato, T. , Nakamura, A. , Hatano, K. , Nagumo, S. , Kubota, K. , Fukuda, H. , Ito, K. , & Kojima, S. (2001). Neural substrates for recognition of familiar voices: A PET study. Neuropsychologia, 39(10), 1047–1054. DOI: 10.1016/S0028-3932(01)00037-9 11440757

[bib72] Newman, R. S. , & Evers, S. (2007). The effect of talker familiarity on stream segregation. Journal of Phonetics, 35(1), 85–103. DOI: 10.1016/j.wocn.2005.10.004

[bib73] Norris, D. , McQueen, J. M. , & Cutler, A. (2003). Perceptual learning in speech. Cognitive Psychology, 47(2), 204–238. DOI: 10.1016/S0010-0285(03)00006-9 12948518

[bib74] Nygaard, L. C. , & Pisoni, D. B. (1998). Talker-specific learning in speech perception. Perception and Psychophysics, 60(3), 355–376. DOI: https://doi.org/10.3758/BF03206860, PMID: 9599989 9599989 10.3758/bf03206860

[bib75] Perrachione, T. K. , Pierrehumbert, J. B. , & Wong, P. C. M. (2009). Differential neural contributions to native- and foreign-language talker identification. Journal of Experimental Psychology: Human Perception and Performance, 35(6), 1950–1960. DOI: https://doi.org/10.1037/a0015869, PMID: 19968445, PMCID: PMC2792570 19968445 10.1037/a0015869 PMC2792570

[bib76] Perrachione, T. K. , & Wong, P. C. M. (2007). Learning to recognize speakers of a non-native language: Implications for the functional organization of human auditory cortex. Neuropsychologia, 45(8), 1899–1910. DOI: https://doi.org/10.1016/j.neuropsychologia.2006.11.015, PMID: 17258240 17258240 10.1016/j.neuropsychologia.2006.11.015

[bib77] Perrodin, C. , Kayser, C. , Abel, T. J. , Logothetis, N. K. , & Petkov, C. I. (2015). Who is that? Brain networks and mechanisms for identifying individuals. Trends in Cognitive Sciences, 19(12), 783–796. DOI: https://doi.org/10.1016/j.tics.2015.09.002, PMID: 26454482, PMCID: PMC4673906 26454482 10.1016/j.tics.2015.09.002 PMC4673906

[bib78] Peterson, G. E. , & Barney, H. L. (1952). Control methods used in a study of the vowels. The Journal of the Acoustical Society of America, 24(2), 175–184. DOI: 10.1121/1.1906875

[bib79] Poeppel, D. (2003). The analysis of speech in different temporal integration windows: Cerebral lateralization as “asymmetric sampling in time.” Speech Communication, 41(1), 245–255. DOI: 10.1016/S0167-6393(02)00107-3

[bib80] Rauschecker, J. P. , & Scott, S. K. (2009). Maps and streams in the auditory cortex: Nonhuman primates illuminate human speech processing. Nature Neuroscience, 12(6), 718–724. DOI: https://doi.org/10.1038/nn.2331, PMID: 19471271, PMCID: PMC2846110 19471271 10.1038/nn.2331 PMC2846110

[bib81] Robin, D. A. , Tranel, D. , & Damasio, H. (1990). Auditory perception of temporal and spectral events in patients with focal left and right cerebral lesions. Brain and Language, 39(4), 539–555. DOI: 10.1016/0093-934X(90)90161-9 2076495

[bib82] Rogers, J. C. , & Davis, M. H. (2018). Inferior frontal cortex contributions to the recognition of spoken words and their constituent speech sounds. Journal of Cognitive Neuroscience, 29(5), 919–936. DOI: https://doi.org/10.1162/jocn_a_01096, PMID: 28129061, PMCID: PMC6635126 10.1162/jocn_a_01096 PMC6635126 28129061

[bib83] Ross, L. A. , McCoy, D. , Wolk, D. A. , Branch Coslett, H. , & Olson, I. R. (2010). Improved proper name recall by electrical stimulation of the anterior temporal lobes. Neuropsychologia, 48(12), 3671–3674. DOI: https://doi.org/10.1016/j.neuropsychologia.2010.07.024, PMID: 20659489 20659489 10.1016/j.neuropsychologia.2010.07.024

[bib84] Roswandowitz, C. , Kappes, C. , Obrig, H. , & Von Kriegstein, K. (2018). Obligatory and facultative brain regions for voice-identity recognition. Brain, 141(1), 234–247. DOI: https://doi.org/10.1093/brain/awx313, PMID: 29228111, PMCID: PMC5837691 29228111 10.1093/brain/awx313 PMC5837691

[bib85] Schall, S. , Kiebel, S. J. , Maess, B. , & von Kriegstein, K. (2014). Voice identity recognition: Functional division of the right STS and its behavioral relevance. Journal of Cognitive Neuroscience, 27(2), 280–291. DOI: https://doi.org/10.1162/jocn_a_00707, PMID: 25170793 10.1162/jocn_a_00707 25170793

[bib86] Schmidt, G. L. , DeBuse, C. J. , & Seger, C. A. (2007). Right hemisphere metaphor processing? Characterizing the lateralization of semantic processes. Brain and Language, 100(2), 127–141. DOI: https://doi.org/10.1016/j.bandl.2005.03.002, PMID: 17292739 17292739 10.1016/j.bandl.2005.03.002

[bib87] Schwartz, J. , & Tallal, P. (1980). Rate of acoustic change may underlie hemispheric specalization for speech perception. Science, 207(4437), 1380–1381. DOI: https://doi.org/10.1126/science.7355297, PMID: 7355297 7355297 10.1126/science.7355297

[bib88] Scott, S. K. , Blank, C. C. , Rosen, S. , & Wise, R. J. S. (2000). Identification of a pathway for intelligible speech in the left temporal lobe. Brain, 123(12), 2400–2406. DOI: https://doi.org/10.1093/brain/123.12.2400, PMID: 11099443, PMCID: PMC5630088 11099443 10.1093/brain/123.12.2400 PMC5630088

[bib89] Siegal, M. , Carrington, J. , & Radel, M. (1996). Theory of mind and pragmatic understanding following right hemisphere damage. Brain and Language, 53(1), 40–50. DOI: https://doi.org/10.1006/brln.1996.0035, PMID: 8722898 8722898 10.1006/brln.1996.0035

[bib90] Souza, P. , Gehani, N. , Wright, R. , & McCloy, D. (2013). The advantage of knowing the talker. Journal of the American Academy of Audiology, 24(8), 689–700. DOI: https://doi.org/10.3766/jaaa.24.8.6, PMID: 24131605, PMCID: PMC3801269 24131605 10.3766/jaaa.24.8.6 PMC3801269

[bib91] Stevens, A. A. (2004). Dissociating the cortical basis of memory for voices, words and tones. Cognitive Brain Research, 18(2), 162–171. DOI: https://doi.org/10.1016/j.cogbrainres.2003.10.008, PMID: 14736575 14736575 10.1016/j.cogbrainres.2003.10.008

[bib92] Theodore, R. M. , & Monto, N. R. (2019). Distributional learning for speech reflects cumulative exposure to a talker’s phonetic distributions. Psychonomic Bulletin and Review, 26(3), 985–992. DOI: https://doi.org/10.3758/s13423-018-1551-5, PMID: 30604404, PMCID: PMC6559869 30604404 10.3758/s13423-018-1551-5 PMC6559869

[bib93] Tranel, D. , Damasio, H. , & Damasio, A. R. (1997). A neural basis for the retrieval of conceptual knowledge. Neuropsychologia, 35(10), 1319–1327. DOI: 10.1016/S0028-3932(97)00085-7 9347478

[bib94] Turkeltaub, P. E. , & Branch Coslett, H. (2010). Localization of sublexical speech perception components. Brain and Language, 114(1), 1–15. DOI: https://doi.org/10.1016/j.bandl.2010.03.008, PMID: 20413149, PMCID: PMC2914564 20413149 10.1016/j.bandl.2010.03.008 PMC2914564

[bib95] van der Burght, C. L. , Goucha, T. , Friederici, A. D. , Kreitewolf, J. , & Hartwigsen, G. (2019). Intonation guides sentence processing in the left inferior frontal gyrus. Cortex, 117, 122–134. DOI: https://doi.org/10.1016/j.cortex.2019.02.011, PMID: 30974320 30974320 10.1016/j.cortex.2019.02.011

[bib96] Van Lancker, D. R. (1980). Cerebral lateralization of pitch cues in the linguistic signal. Papers in Linguistics, 13(2), 201–277. DOI: 10.1080/08351818009370498

[bib97] Van Lancker, D. R. , & Canter, G. J. (1982). Impairment of voice and face recognition in patients with hemispheric damage. Brain and Cognition, 1(2), 185–195. DOI: 10.1016/0278-2626(82)90016-1 6927560

[bib98] Van Lancker, D. R. , & Kreiman, J. (1987). Voice discrimination and recognition are separate abilities. Neuropsychologia, 25(5), 829–834. DOI: 10.1016/0028-3932(87)90120-5 3431677

[bib99] von Kriegstein, K. , Eger, E. , Kleinschmidt, A. , & Giraud, A. L. (2003). Modulation of neural responses to speech by directing attention to voices or verbal content. Cognitive Brain Research, 17(1), 48–55. DOI: 10.1016/S0926-6410(03)00079-X 12763191

[bib100] von Kriegstein, K. , & Giraud, A. L. (2004). Distinct functional substrates along the right superior temporal sulcus for the processing of voices. NeuroImage, 22(2), 948–955. DOI: https://doi.org/10.1016/j.neuroimage.2004.02.020, PMID: 15193626 15193626 10.1016/j.neuroimage.2004.02.020

[bib101] von Kriegstein, K. , Smith, D. R. , Patterson, R. D. , Kiebel, S. J. , & Griffiths, T. D. (2010). How the human brain recognizes speech in the context of changing speakers. Journal of Neuroscience, 30(2), 629–638. DOI: https://doi.org/10.1523/JNEUROSCI.2742-09.2010, PMID: 20071527, PMCID: PMC2824128 20071527 10.1523/JNEUROSCI.2742-09.2010 PMC2824128

[bib102] Wada, J. , & Rasmussen, T. (1960). Intracarotid injection of sodium amytal for the lateralization of cerebral speech dominance: Experimental and clinical observations. Journal of Neurosurgery, 17(2), 266–282. DOI: 10.3171/jns.1960.17.2.0266 17564192

[bib103] Wernicke, C. (1874). Der aphasische Symptomencomplex: Eine psychologische Studie auf anatomischer Basis. Cohn.

[bib104] Xie, X. , & Myers, E. B. (2018). Left inferior frontal gyrus sensitivity to phonetic competition in receptive language processing: A comparison of clear and conversational speech. Journal of Cognitive Neuroscience, 30(3), 267–280. DOI: https://doi.org/10.1162/jocn_a_01208, PMID: 29160743 29160743 10.1162/jocn_a_01208 PMC8048105

[bib105] Yi, H. G. , Leonard, M. K. , & Chang, E. F. (2019). The encoding of speech sounds in the superior temporal gyrus. Neuron, 102(6), 1096–1110. DOI: https://doi.org/10.1016/j.neuron.2019.04.023, PMID: 31220442, PMCID: PMC6602075 31220442 10.1016/j.neuron.2019.04.023 PMC6602075

[bib106] Zäske, R. , Awwad Shiekh Hasan, B. , & Belin, P. (2017). It doesn’t matter what you say: fMRI correlates of voice learning and recognition independent of speech content. Cortex, 94, 100–112. DOI: https://doi.org/10.1016/j.cortex.2017.06.005, PMID: 28738288, PMCID: PMC5576914 28738288 10.1016/j.cortex.2017.06.005 PMC5576914

[bib107] Zatorre, R. J. , & Belin, P. (2001). Spectral and temporal processing in human auditory cortex. Cerebral Cortex, 11(10), 946–953. DOI: https://doi.org/10.1093/cercor/11.10.946, PMID: 11549617 11549617 10.1093/cercor/11.10.946

[bib108] Zatorre, R. J. , Meyer, E. , Gjedde, A. , & Evans, A. C. (1996). PET studies of phonetic processing of speech: Review, replication, and reanalysis. Cerebral Cortex, 6(1), 21–30. DOI: https://doi.org/10.1093/cercor/6.1.21, PMID: 8670635 8670635 10.1093/cercor/6.1.21

